# District nurses’ views on and experiences with a telemedicine educational programme in palliative care

**DOI:** 10.1111/scs.12818

**Published:** 2020-01-15

**Authors:** Eithne Hayes Bauer, Georg Bollig, Karin B. Dieperink

**Affiliations:** ^1^ Medical Department University Hospital of Southern Jutland Aabenraa Denmark; ^2^ IRS‐centre South Jutland University of Southern Denmark Palliative Team and Department for Palliative Care Medical Department University Hospital of Southern Jutland Aabenraa Denmark; ^3^ Department of Oncology Odense University Hospital Odense C Denmark; ^4^ Department of Clinical Research University of Southern Denmark Odense Denmark

**Keywords:** qualitative study, focus groups, district nurses, telemedicine, telehealth education, palliative care, perspective

## Abstract

**Background:**

Medical advancements, limited resources and shifting demographics have increased the number of patients with palliative care needs in primary care. To address educational needs, the specialised palliative care team of South Jutland, Denmark, created a telemedicine educational programme in palliative care to empower district nurses.

**Aim:**

The study aimed to explore district nurses’ views on and experiences with a telemedicine educational programme in palliative care.

**Research methods:**

A qualitative explorative study based on interpretive description was conducted. Data collection consisted of four focus group interviews with district nurses from three municipalities, supplemented by participant observations and a focus group interview with teachers from the specialised palliative care team. Data were analysed using predominately inductive thematic analysis.

**Results:**

District nurses (n = 15) who participated in the programme and members of the specialised palliative care team (n = 6) who taught the programme were included. Analysis revealed the following advantages: reaffirming and updating existing knowledge, reduced professional isolation and creation of a forum to promote knowledge dissemination. A disadvantage was limited interaction between teachers and district nurses, questioning suitability for teaching complex palliative care. Initial technical problems affected motivation to participate. Organisational support differed between participating municipalities resulting in varying degrees of programme integration. Despite advantages of IT‐expert‐led sessions, key‐nurse‐led sessions in smaller groups proved more beneficial, suggesting a combination of IT support and key‐nurse management to maximise benefits.

**Conclusion:**

The use of an inter‐professional telemedicine educational programme to teach palliative care to district nurses is beneficial. However, programmes should be designed for interactivity and address varying educational needs. Key‐nurse roles require managerial and IT support to optimise knowledge dissemination. Further research on implementation of telemedicine education in palliative care is needed.

## Introduction

The World Health Organisation (WHO) and current research are attempting to integrate telemedicine into healthcare systems – including the area of palliative care 1, 2, 3, 4, 5. In accordance with WHO, the Danish Health Authority redefined palliation to include *all* patients suffering from life‐threatening illnesses 6, 7. Implied consequences indicate greater demands for palliative care services and education especially in the primary care sector. Qualitative studies related to district nurses’ experiences with telemedicine educational programmes in palliative care are limited, and a literature search performed in PubMed and CINAHL failed to retrieve relevant results. However, a quantitative study by Ray *et al.,* 8 in rural Australia, revealed that all participants (n = 101) in a palliative telemedicine educational programme found content useful, with significant changes in confidence in provision of care. Results from the Highland Hospice Project Echo in Scotland are also positive for teaching rural healthcare professionals’ palliative care via telemedicine 9. Participants described increased knowledge, confidence and feelings of connectedness and support 9. Traceable results for the use of inter‐professional educational telemedicine on a larger scale were also scarce but with some results from North and South America 10, 11, 12. Although not specific to palliative care, these studies revealed perceptions of primary care workers and factors affecting sustainability including time constraints, professional rural isolation, continued learning and convenience 10, 11, 12. Despite implied advantages, distance learning courses cannot exclusively lead to changes in work practice; interaction and communication of knowledge between stakeholders are needed to transform professional practices 11. With increasing demands for palliative care provision in primary care, the need to expand knowledge in this area is growing. Some information exists on the use of telemedicine for educating healthcare professionals in primary care, but very little is known about the education of district nurses in palliative care via telemedicine and their perception of the same. Therefore, the purpose of this study was to explore district nurses’ views on and experiences with a telemedicine educational programme in palliative care and whether they experienced empowerment through participation in the programme. This study aims to contribute to knowledge on inter‐professional educational telemedicine in palliative care and inform implementation of existing programmes.

## Methods

A qualitative explorative study was performed based on interpretive description. Interpretive description acknowledges subjective and experiential knowledge as fundamental sources of applied practice and insight and that human experiences are constructed within a social context to which they are connected 13. The current study is situated within a larger project concerning telemedicine in palliative care in South Jutland, Denmark 14. By conducting focus group interviews and participant observations, district nurses’ and specialised palliative care team (SPCT) teachers’ experiences, shared and individual, are described and interpreted. Combinations of methods and data triangulation are used to broaden the scope of the sample adding contextual depth to data and strengthening subsequent findings 15.

### Telemedicine educational programme set‐up

Using Cisco WebEx, a closed‐circuit synchronous link, SPCT teachers including physicians, nurses, social workers, physiotherapists and psychologists broadcasted monthly lectures to four municipalities in South Jutland, Denmark. All district nurses (n = 333) were invited to attend. Dates and times were planned in advance ensuring maximum attendance. Palliative care is divided into basic and specialised palliative care as outlined in Box 1. Topics covered basic palliative care and included acupuncture, shortness of breath, pain, antibiotic treatment, depression, constipation, delirium and grief, loss and pain. Participants were encouraged to submit cases for use in the programme. Sessions commenced with a greeting to ensure participants were logged on, followed by a 30‐minute lecture and ended with questions and answers conducted via a chat function. Slides used during transmission were distributed via email.

Box 1 **Basic palliative care vs. specialised palliative care**

**Table:** 

Palliative care consists of basic and specialised palliative care, depending on knowledge and skills necessary for caring for patients with life‐threatening illnesses. Knowledge of basic palliative care is required of all healthcare professionals, where palliative care is not their main task but may come into contact with patients with life‐threatening illnesses. In contrast, palliative care specialists have palliation as their main task, working daily with patients with complex palliative care needs in multidisciplinary teams 6. The Danish Health Authority recommends that SPCTs promote knowledge in palliative care by teaching basic palliative care to healthcare professionals 6. This in line with recommendations from the European Association on Palliative Care for inter‐disciplinary learning in the core competencies of palliative care 16.

### Data collection

#### Focus groups

Four focus group interviews were conducted to explore common experiences and differing opinions through group discussions, including a pilot interview to validate the interview guide 17, 18. Focus group interviews are valuable in relation to internal evaluation, educational projects and educational experiences in combination with other methods or alone 18. Therefore, focus groups are found suitable in gaining an understanding of collective normative practices based on views and experiences of district nurses*.* The interview guide, which contained open questions, required minimal changes following a pilot interview. Therefore, the pilot interview forms part of the data set. A further focus group interview with SPCT teachers was included, to triangulate data. An observer was present during all focus group interviews, and the first author held the role of moderator.

#### Participant observations

Two participant observations were performed to gain insight into the culture surrounding sessions: one in a receiving area amongst district nurses and one in an SPCT office during transmission of a telemedicine session 19. Field notes were recorded during observations.

### Participants

District nurses who took part in the programme were invited to participate in the study via e‐mails from nursing managers acting as gatekeepers. The gatekeepers were also representatives from the four municipalities in the research project’s steering group 14. A sample was recruited aiming for maximum variation for age, municipality, postgraduate education, years of experience in health care and years of experience in palliative care. Information was gathered to illustrate participants’ characteristics in relation to findings. Inclusion criteria were district nurses employed in one of the four municipalities, who participated at least once in the programme. Despite efforts to include participants from all four municipalities, one municipality chose not to participate due to resource issues. The total number of participants in the telemedicine educational programme is unknown, as no record of participation was maintained at the locations where nurses met to participate. Group size varied from approximately 5–30 nurses per participating group. Each municipality consisted of 2–5 groups.

### Ethical considerations

Ethical approval was sought together with ethical approval for the larger project by Bollig *et al.,* 14 from the Regional Ethical Committee (S‐20172000), and has been reported to the Danish Data Protection Agency (2008‐58‐0035). This study complies with WMA’s Declaration of Helsinki 20. Participation was voluntary. Written and oral information was provided prior to participation 21. Written consent was obtained from focus group participants, who were informed of the possibility of withdrawing consent without further consequences 21. Participants were anonymised following transcription and encouraged to maintain confidentiality of group discussions 21. SharePoint was used for data storage. Demographic data were removed from Table 2 to protect the anonymity of participants from the SPCT.

### Data analysis

The first author transcribed all focus group interviews. Data comprised of two data sets and were analysed by all authors using interpretive description following the steps outlined in Box 2 18, 22. Results are reported in accordance with a checklist for consolidated criteria for reporting qualitative research 23.

Box 2 **Steps in interpretative descriptive analysis of district nurses’ experiences with a telemedicine educational programme in palliative care**

**Table:** 

Immersion in data was gained by reading, rereading and listening to audio recordings of data. Initial coding was performed loosely and iteratively, zooming in and out of the data in order to see patterns and avoid premature closure Themes and patterns were derived and tested guided by the following questions; ‘Why is this here? Why not something else? What does it mean?’ All three authors participated in data analysis, Themes and patterns were actively ordered into a thematic summary in a meaningful and coherent manner making sense of the research question so that it can be understood in the applied practice. An openness to critique and an awareness of the tentative nature of results are transparent in the analytic process in order to achieve credibility.

### Theoretical background

The study’s theoretical background draws on theory from continuing education for healthcare professionals. Ferris *et al.* 24 suggest that knowledge acquisition alone is insufficient in bringing about changes in clinical practice. Instead, a cascade of steps including attitude, knowledge, skills, behaviour, patient/family experience and societal experience is necessary. To incorporate these steps into the applied field, Ferris *et al.* highlight the following;

1. Tailoring messages to suit the needs and resources of the people for whom they are intended.
2. Prioritising and supporting mentoring of students in applied fields to enhance mastering of skills.
3. Ensuring organisational endorsement to enable and foster desired outcomes 24.

The lens provided by the above‐mentioned theory will be applied in discussion of findings.

### Reflexivity

Interpretive description acknowledges the author’s background in clinical practice allowing one to draw on practical experience in addition to empirical and theoretical perspectives 25. The first author has experience from tertiary and primary care settings, but no prior experience of telemedicine. The first author’s relationship with study participants is limited to the role of researcher.

## Results

Four focus group interviews with district nurses (n = 15), after which data saturation was achieved, and one focus group interview with SPCT teachers (n = 6) were performed. Table 1 below outlines participant demographics, linking participants numerically to results. Ages ranged from 31 to 61, with an average of 47.5 years. Most had over 5 years’ work experience. Session participation ranged from 1 to 5 from May 2018 to December 2018. One participant had taken part in over 5 sessions; all others participated in 1–5.

**Table 1:** Demographics of district nurses and SPCT^a^ members who participated in a study about a telemedicine educational programme in palliative care

Participants DN^b^/SPCT^c^ N = 21	Age mean 47.5 years	Gender f/m	Education	Experience in years as healthcare professional	Experience in years in palliative care	Frequency of contact with palliative patients
1 DN	41	F	BScN^d^ + PG^e^	>5 years	6 years	Weekly
2 DN	54	F	RN + PG	>5 years	16 years	Weekly
3 DN	54	F	RN + PG	>5 years	16 years	Weekly
4 DN	59	F	RN + PG	>5 years	30 years	Weekly
5 DN	43	F	RN + PG	>5 years	16 years	Daily
6 DN	59	F	BScN + PG	>5 years	1 year	Daily
7 DN	61	F	RN	>5 years	35 years	Daily
8 DN	44	F	RN + PG	>5 years	20 years	Daily
9 DN	54	F	RN	>5 years	30 years	Daily
10 DN	56	F	RN + PG	>5 years	33 years	Daily
11 DN	37	F	BScN + PG	>5 years	13 years	None
12 DN	34	F	BScN + PG	>5 years	6 years	Daily
13 DN	31	F	BScN	<5 years	1 year	Daily
14 DN	40	F	BScN	<5 years	2 years	Daily
15 DN	45	F	BScN	>5 years	3 years	Daily
16 SPCT				<5 years		Daily
17 SPCT				>5 years		Daily
18 SPCT				>5 years		Daily
19 SPCT				>5 years		Daily
20 SPCT				>5 years		Daily
21 SPCT				>5 years		Daily

a Certain demographics are removed to protect SPCT’s anonymity.

b District nurse.

c Specialised palliative care team.

d Bachelor of Science in Nursing.

e Postgraduate education.

Four themes emerged from the data: teaching methods in palliative care, expectations and adaptations, organisational set‐up and technology.

The four themes are presented below; subthemes are summarised followed by examples from data. An example of the analysis is included in Table 2.

**Table 2:** Example of predominately inductive thematic analysis of district nurses’ experiences with a telemedicine educational programme in palliative care

Main theme	Category	Subcategory	Code	Citations
Palliative care and teaching method	Limitations and possibilities	limited method, little interaction, Direct route, reaffirm skills	tutorial, interaction, direct, reconfirming	‘Well, one of the disadvantages there are, is that you can’t sort of interact with the other participants, so you can’t just have a chat about how you manage this situation’. (participant no. 11)
	Increasing familiarity	Familiarity with SPCT, knowing who to contact	Get to know, Contact SPCT	‘You put a face on for example the palliative team and that can make it easier for some to think; “eh, I better call them, or write, or something”’ (participant no. 2)
	Case‐based content	Increase relevance to relate to daily practice, unaware of options, reasons for submitting cases	Cases, influence on content, Relevance, Lack of time, irrelevance	‘These are the situations we’re standing in. So, it could be good with some cases; “what do we do if we’re in this [sic.situation]” and an understanding for each other’. (participant no. 14)
	Two states of mind	Face‐to‐face vs. telemedicine, two methods and two states of mental awareness	Focus, concentration, short and concise, multitasking, formal and informal	‘So we’re all there with the purpose of learning. With the other, we’re just there, yeah, maybe we can sit and eat lunch;” no yeah, there was something or other they wanted to tell us about today”. Well, there’s a different awareness’. (participant no. 2)

### Teaching methods in palliative care

#### Possibilities and limitations

Educational telemedicine provided possibilities and limitations in teaching palliative care. District nurses experienced increased self‐assurance in their knowledge base. Knowledge dissemination directly from experts was valued for strengthening validity and removing information loss. All participants found topics relevant to clinical practice, although several found the level taught too low, lacking interaction and with minimal case‐based content.

Experienced district nurses expressed renewed self‐assurance in their knowledge base when information relayed by experts corresponded to their knowledge.

New knowledge, I don’t think we got that, but we got an assurance in that what we actually do, is also what they recommend in there, so that’s been good. (Participant no. 12)

In comparison with face‐to‐face, the method was reported as top‐down. Several participants described dissatisfaction with limited interaction but valued direct and simultaneous information to all four municipalities.

Critique of the method was echoed by SPCT teachers who described challenges in teaching via telemedicine, as ability to gauge participants’ responses was lost. This led to adoption of a didactic approach based on minimal interaction;

We’ve all been used to having a little more interaction instead of having such an old‐ fashioned tutorial and it becomes more tutorial‐like. When you’re sitting there in front of the screen and are limited to that. (Participant no. 19)

An awareness of limitations and attempts to counteract these evolved but remained insufficient. Participants proposed ways to reduce top‐down approaches: increased interaction, further training for teachers, varying teaching methods, increasing quality of visual presentations and using case presentations, which were supported by findings from SPCT teachers.

Despite participants’ desires for relatable case‐based content, several were unaware of the possibility of submitting cases.

I’m not actually sure if any of us have sent an actual case in to the programme. I’m actually thinking; ‘Did I know about that?’ (Participant no. 11)

Reasons for not submitting cases were described as follows: work pressures, lack of motivation and fear of irrelevance to others. Participants proposed mandatory case submission.

#### Two states of mind, two methods: educational telemedicine vs. face‐to‐face

In comparison with face‐to‐face teaching methods, many participants found that reduced interaction affected their focus on the topic. Although aware of advantages of educational telemedicine, such as time‐saving and cost‐effectiveness, many participants expressed a preference for face‐to‐face methods. Reasons given included increased interaction, animation and focus on content. Face‐to‐face methods were described as possessing quality, whereas telemedicine was described as effective. As to why the methods were perceived differently became apparent during analysis. Participants described states of mind they entered into when participating in telemedicine or face‐to‐face encounters.

I think that it’s really effective. An effective way of doing things, with screen courses, but I think – quality wise – I think for me it would be better to be invited on a course. Where I was there, and my focus was there, because one is invited there. (Participant no. 2)

Being ‘invited’ suggests formality. One participant described the difference in relation to being on a course, as a different mental awareness, when participating physically removed from one’s workplace. In contrast, the state of mind entered into in telemedicine sessions was described as relaxed, comfortable, informal, governed by the day’s work and requiring little mental preparation. Participants described less focus on the topic and reduced interaction, which led to reflection on subject matter; topics covered should fit the method. Several described preferences for short and concise subject matter which was considered feasible during a busy working day.

### Expectations and adaptations

#### Renewed focus on palliative care and reduced professional isolation

Topics covered during sessions led to a renewal of participants focus on palliative care, raising the competency of some and reducing professional isolation.

Concrete examples of application of knowledge gained from the programme were few but indicate translation of knowledge into practice.

I have just been in something with a palliative patient, who had mouth‐dryness where, she said in one of the sessions eh; “Use ordinary food oil with lemon zest”. I don’t know why that stuck, and it works and that’s good. (Participant no. 15)

Younger district nurses described independence and self‐reliance as necessary for providing palliative care in the primary sector. They also described a lack of professional sparring leading to feelings of professional isolation. They described situations in which they were unsure of how to handle, where they drew on information from the telemedicine sessions. They expressed a wish for the possibility of using telemedicine in the field to access expert advice when dealing with complex situations

If I’m out at a patient that I can just call the palliative team with a screen; “yeah, I’m out at so and so patient’s [sic. house]. I have these problems. What do you think?” That you get that professional response straight away. (Participant no. 15)

#### Changed behaviour in learning situation

Several participants experienced changes in the manner in which they participated in educational encounters. For some, the altered behaviour suited their learning style enhancing concentration. Others became passive. Participants reflected on the importance of seeking clarification. Despite encouragement from SPCT teachers to ask questions, the majority experienced suppressing questions.

…that amount of people – we’re 25–30 people … then we’re finished. And an unrest already begins, that has an effect on whether one thinks; “is it very important for me to ask this question?” (Participant no. 2)

Reasons for not asking included lack of direct interaction, not wanting to interrupt, irrelevance of questions to others, poor typing skills, difficulty expressing oneself in writing and reluctance to delay colleagues in resuming work. Participants described the chat function as difficult to use. This finding was irrespective of organisational set‐up, although most prevalent in larger groups.

#### Dual focus during sessions

A recurring aspect during telemedicine sessions was difficulty in maintaining concentration, as a result of competing tasks. Participants were divided between concentrating on sessions and being at work. Tasks such as answering telephones, documenting or eating lunch were performed alongside participation in sessions. Several participants reported workloads remaining unchanged, despite efforts to reduce visits on days scheduled for telemedicine sessions. Some reported missing out, while others arrived late. Participants described how not documenting during sessions could lead to overtime creating a dual focus during sessions.

I lose focus sometimes. I can be distracted by a telephone ringing, or; “hey! I have to remember to document about where I just came from, and nobody will notice if I just sit here and write”. (Participant no. 14)

For those manning an acute telephone, desire to participate and fear of missing out were described as more important than interrupting sessions. Others described how a designated nurse took calls and performed miscellaneous tasks, while colleagues participated in sessions. Difficulties in concentration with disturbances leading to visible distractions were found in field observations.

A lot is going on at once around the table, as lunches are consumed, others arrive and pull out chairs. Sounds from a microwave, cups and plates, cutlery, whispering voices, plastic bags and paper compete with (…)’s voice, making it difficult to concentrate and follow the lecture. (Observations from field study)

Several participants proposed recording sessions for convenient participation. SPCT members expressed a wish to podcast sessions to increase and ease participation.

Basically, my wish is that we can podcast, so people can download it whenever they want to. (Participant no. 20)

### Organisational set‐up

During focus group interviews, information emerged about the telemedicine set‐ups employed by the three municipalities. Through analysis, it was possible to discern barriers and facilitators apparent in each, as outlined in Table 3 below. *Key nurse* refers to nurses managing telemedicine sessions.

**Table 3:** The set‐up in three municipalities participating in a study on a telemedicine educational programme in palliative care

	Municipality A	Municipality B	Municipality C
Responsibility for set‐up	IT‐expert + manages chat	Key nurse in palliation + manages chat	Key nurse sets up session. Participants manage chat
Locations joining sessions	two out of five possible locations	Five out of five possible locations	One location alternating between two out of three possible locations
Participants	20–30 nurses from different districts	5–8 nurses in own districts	15–25 nurses from different districts
Barriers	Reluctant to ask questions due to large numbers of participants. Lack of group discussions after sessions	Responsible for set‐up. Lack of technological support stressful. Lack of network outside of own district. Reduced focus on content	Lack of technological support during sessions. Distance to location can hinder participation. Difficulty planning participation. Reduced focus on content
Facilitators	Focus on content. Possibility to network with nurses from other districts. Not responsible for set‐up	Development of technological skills. Group discussions and questions and answers following sessions. Invitation to allied healthcare professionals to participate in sessions	Possibility to network with nurses from other districts. Not responsible for set‐up

#### Group size and key‐nurse role

District nurses described familiarity and informality associated with participation in sessions in one’s own group. Key‐nurse‐led postsession group discussions furthered knowledge dissemination in palliative care. Participants described how topics from sessions were discussed and related directly to clinical practice.

And use the knowledge we just received and say; “is there something here we should be particularly aware of with the clients we are visiting or where there is the need for it?” (Participant no. 4)

This benefit was not apparent in larger IT‐expert‐led sessions. In contrast, participants described rushing back to work, reluctant to detain colleagues by asking questions.

### Technology

#### Frustration over technical problems

Technical problems affected concentration and motivation. Despite mastering necessary skills, participants found the system overly complicated. Technical problems were prevalent during the first 2 months and related to lack of IT support or skills in key nurses and SPCT members. Regardless of cause, technical problems resulted in diminished concentration amongst participants.

I’ve sat and thought; ‘I could just as well go in and do something else, because I’m not going to get anything out of this!’ It’s such a shame, because it’s such a good initiative, but all of the trouble with that, so focus; it vanishes. (Participant no. 12)

Some district nurses described sessions as time mismanagement and stopped participating until problems were resolved, while others continued participating and assigned problems encountered to teething problems, which were adequately resolved.

## Discussion

This study aimed to explore a telemedicine educational programme in palliative care from district nurses’ perspective, by describing their experiences and views. Main findings were as follows:

- District nurses found the programme beneficial in confirming and updating their knowledge base.
- The programme led to renewing focus on topics in palliative care and reducing professional isolation.
- The participating district nurses reported clinical relevance, with some examples of application of knowledge gained. However, lack of interaction restricted teaching methods, reducing the methods’ aptness for teaching complex palliative care.
- Session set‐ups differed in municipalities. Through comparison, the most optimal set‐up could be derived. Key‐nurse‐led sessions in smaller groups contributed positively to further knowledge dissemination through postsession group discussions. This should be addressed in the future to improve learning.
- Motivation for participation was related to content, competing tasks and technological support.

### Discussion of participants

Difficulty in recruiting younger participants warranted a further focus group interview, after which no new data emerged. Therefore, included participants were deemed representative. Participants’ high average age – 47.5 years – is a reflection of a tendency amongst district nurses in Denmark, whose estimated average age is 46.5 years 26, thus increasing transferability of findings in a Scandinavian primary healthcare context.

### Telemedicine, technology and future proposals

Educational telemedicine required a period of adaptation for district nurses and SPCT teachers. Some key nurses acquired increased skills, displaying perseverance and innovation in handling initial technical problems. For others, the system proved overly complicated and technical problems remained unresolved, affecting motivation to participate. An important factor, as indicated here, is recognising the need for technical skills as a prerequisite for participation in the programme. According to Ferris et al., supporting mentoring in the applied field to enhance skills is central to continued education 24. Therefore, providing training prior to participation and in the field for district nurses and SPCT teachers can increase motivation for participation. Furthermore, Paul *et al.* 10 suggest that designing telemedicine educational programmes for convenience and utilising straightforward technology contribute to programme sustainability. According to Wong *et al*., 27 a fit between course technology and participants’ needs and priorities is necessary. While bearing in mind the need for data protection, avoiding complicated technology, choosing user‐friendly systems and resolving technical problems promptly are important for maintaining motivation and should be considered a design priority in telemedicine educational programmes.

Participants described varying expectations to programme content and teaching methods; some were met, whereas others required changes. Adapting to the method involved a change in teaching approach for SPCT teachers and a change in learning style for district nurses. According to Paul *et al.,* 10 understanding the underlying philosophy in educational telemedicine as a method of remodelling health care by departing from healthcare provision to increasing local capacity is an important motivational factor in entering into sessions with an attitude conducive to learning. This is in line with Ferris *et al.,* 24 who states that an attitude conducive to learning precedes knowledge acquisition. Directing attention towards a common understanding of programme aims and desired outcomes may prove beneficial during a period of adaptation. In addition, training tutors to teach via telemedicine, incorporating skilful presentations and variation in teaching methodology, may increase motivation to participate and ease adaptation. Incorporating education theory, such as Kolb’s experiential learning theory, which is found effective amongst nurses, could provide a framework for inter‐professional educational telemedicine programmes 28, 29.

District nurses reported altered behaviour during participation in sessions. For some, technology represented an opportunity to learn, increasing focus on palliative care, reminiscent of learning environments from tertiary settings and reducing professional isolation. This finding was widely supported in literature, which, in addition, states that reducing professional isolation contributes to employee retention 8, 9, 10. For others, participation represented time mismanagement, as competing tasks outweighed the benefits of participation. Lack of motivation pertaining to technological problems, content and competing tasks proved a barrier for several district nurses – a finding reverberated by Pereira *et al.,* and Munoz and Bradley 9, 11. According to Ferris, recognising practical barriers and tailoring content and design to suit participants’ needs and time constraints, along with managerial endorsement, are important aspects in continued education of healthcare professionals 24. Protected time for participation in sessions and training skills to meet technological requirements are important aspects warranting managerial support 9, 11. Therefore, examining existing barriers, taking concrete steps to reduce these, for example training skills and planning sessions with employees, may promote meaningful participation and limit disruption of competing tasks.

A future proposal voiced by several district nurses and supported by SPCT teachers was the possibility of recording sessions for convenient viewing. Existing literature differs here. On the one hand, availability of prerecorded sessions enables further participation, increasing convenience and flexibility for individual nurses 9. On the other hand, group participation at workplaces improves the possibility of knowledge translation through communal understanding and acceptance of new knowledge 11. As illustrated above, nurses value discussing the educational content in smaller groups following lectures thus strengthening the impact of the educational sessions. Whether viewing prerecorded sessions contributes to meaningful participation remains unclear. However, appreciating the reality of district nurses’ daily challenges and collaborating between actors can facilitate alignment of expectations in relation to programme feasibility and contribute to successful programme design.

### Knowledge gained and future proposals

Renewed focus on palliative care was a direct result of participation in the programme, and all participants reported clinical relevance. Although content was not new for some, participants valued the opportunity to confirm and update existing knowledge, as previously reported 8, 9. Ray *et al.* 8 suggest this as being a result of rural district nurses’ broad experience and pre‐existing knowledge of palliative care. The majority of district nurses had contact with palliative care patients for over 5 years, which may explain why they found the level taught to be low. Ray *et al.* also found that district nurses displayed an interest in case‐based content and stressed the importance of addressing various educational needs 8. Teaching complex palliative care in this study proved challenging, as interaction was limited, which is similar to previous findings 11. Therefore, a major challenge is overcoming limited interaction. The chat function proved insufficient and several participants suppressed the desire to ask questions. In contrast, approaches that employed interaction and enabled networking met educational needs in relation to discussing complex palliative care 8, 9. The importance of interaction is echoed in research, as a collaborative approach between all actors 10. Furthermore, participatory approaches in learning situations aid memory and learning 24. Munoz and Bradley also advocate an inclusive environment facilitating discussions 9. Therefore, substantial efforts should be made to improve interaction in future implementation. The results of this study indicate that nurses require encouragement to participate by asking questions and providing case reports.

A main finding was key‐nurse‐led session’s contribution in smaller groups to further disseminate knowledge in palliative care via postsession group discussions. In the forum created by telemedicine, topics covered during sessions were subsequently discussed, clarifying questions and relating topics to clients. This proved most feasible in smaller key‐nurse‐led groups in comparison with larger IT‐expert‐led groups. Given the complexity of palliative care, it has been shown that the specific programme alone was unable to meet district nurses needs for learning complex palliative care. Nonetheless, through further dissemination, district nurses could translate knowledge into skills reporting an increase in the management of palliative care, where practices ‘*had fallen into place’* following participation in the programme. Although a simple intervention, this practice has not been suggested previously. However, it is supported by recommendations for sustainability of telemedicine educational programmes advocating participation during the working day and in group settings 11. Furthermore, Ferris *et al.* 24 encourage development and support of mentors to ensure translation of knowledge into skills in the applied field. Therefore, supporting the role of key nurses in the forum created via the programme may lead to an increase in desired outcomes and empowerment of district nurses. District nurses with palliative care knowledge could serve as key nurses and moderators.

Derived future proposals are summarised in Table 4 below, the most important of which may be employing user‐friendly interactive solutions, meeting diverse educational needs and supporting key‐nurse‐led sessions.

**Table 4:** Factors to be considered and future proposals in telemedicine educational programmes in palliative care

Related themes	Future proposals
Palliative care teaching methods	Interactive technological solution Assessment of educational needs Vary content to meet diverse needs Mandatory case presentation
Expectations and adaptations	Clear communication of programme aims Participatory approach Telemedicine teacher training Train participants technological skills Postparticipation evaluation Record or podcast sessions
Organisational set‐up	Managerial endorsement Facilitate key‐nurse‐led sessions Promote postsession group discussions Promote workplace participation Promote primary group participation Protected time for participation in sessions Increased employee involvement in planning
Technology	Straightforward user‐friendly technology Prompt resolution of technological problems IT support for key‐nurse‐led sessions

### Strengths and limitations

The limited number of district nurses included in the study may represent a limitation in terms of generalisability. However, use of supplementary methods of data collection strengthens validity of findings.

Use of nursing managers as gatekeepers constitutes a limitation, as one of four municipalities in the programme chose not to participate in the study. Lack of participation in the educational sessions and in the focus group discussions might have been influenced by lack of resources. However, a strength of the study is rigour in relation to sampling of included district nurses, who are found representative of their profession in terms of age, education and experience. Therefore, the impact of lack of participation of one municipality in the study can be seen as limited.

Information on translation of knowledge gained via the programme is based on self‐reported examples and can, therefore, contain recall bias.

### Clinical implications

The use of inter‐professional educational telemedicine has the ability to enhance district nurses’ knowledge and skills in palliative care. To unleash its full potential, several aspects should be addressed in future implementation: increasing interaction, organisational endorsement, district nurses’ diverse educational needs, SPCTs’ training requirements and supporting key nurses’ roles in furthering knowledge dissemination.

Further research is necessary to measure the impact of educational telemedicine on palliative patients’ care. Future studies could include pre‐ and postprogramme observational studies and interviews with patients and relatives to uncover effects of inter‐professional educational telemedicine in palliative care. The use of a model for assessment of telemedicine applications (MAST), which is a framework for evaluating effectiveness and contribution to quality of care, could also be applied to future research to provide valuable in‐depth assessment of telemedicine implementation and use of digital learning in a broader manner 30.

## Conclusion

The purpose of this study was to inform further implementation and increase knowledge in inter‐professional educational telemedicine in palliative care by exploring district nurses’ views and experiences. This study demonstrated that programme content was relevant to clinical practice. Participants experienced increased focus on aspects of palliative care, reduced professional isolation and updated and renewed self‐assurance in their knowledge base. Furthermore, it illustrated that a higher level of interaction is required to address district nurses’ educational needs. Empowerment of district nurses proved difficult to demonstrate as a result of considerable barriers to interaction. Nevertheless, some examples of knowledge transfer exist indicating educational telemedicine’s potential for teaching palliative care. The role of key nurses in postsession group discussions proved a key factor in advancing knowledge dissemination, warranting increased organisational and IT support in future implementation and indeed further research.

## Conflicts of interest

The authors have no conflicts of interest to disclose. The authors alone are responsible for the content and writing of the paper. The paper is the result of a study situated within a larger study by Bollig *et al.* 14.

## Author contributions

Eithne Hayes Bauer devised the study design. Georg Bollig and Karin B. Dieperink reviewed and commented on the study design and suggested modifications. All authors participated in data analysis, reviewed and revised the manuscript critically and participated in discussion of the results. All authors read and approved the final version of the manuscript.

## Ethical approval

Ethical approval was sought together with ethical approval for the larger project by Bollig *et al.,* from the Regional Ethical Committee (S‐20172000), and has been reported to the Danish Data Protection Agency (2008‐58‐0035).

## Funding

The study was unfunded and conducted in association with a master’s dissertation in clinical nursing.
